# Integration of FET PET in neurosurgical resection planning may improve overall survival in patients with newly diagnosed glioblastoma

**DOI:** 10.1093/noajnl/vdag220

**Published:** 2026-08-20

**Authors:** Norbert Galldiks, Philipp Lohmann, Joerg-Christian Tonn

**Affiliations:** Department of Neurology, Faculty of Medicine, University Hospital Cologne, Cologne (N.G.); Institute of Neuroscience and Medicine (INM-3, INM-4), Research Center Juelich, Juelich (N.G., P.L.); Center of Integrated Oncology Aachen Bonn Cologne Duesseldorf (CIO ABCD), Germany (N.G.); Institute of Neuroscience and Medicine (INM-3, INM-4), Research Center Juelich, Juelich (N.G., P.L.); Department of Nuclear Medicine, RWTH Aachen University Hospital, Aachen (P.L.); Department of Neurosurgery, University Hospital Munich, Ludwig-Maximilians-University, Munich (J.-C.T.); German Cancer Consortium (DKTK), Partner Site Munich, Munich (J.-C.T.)

**Keywords:** amino acid, PET-based resection

While neuro-oncological guidelines increasingly advocate for maximal safe resection in newly diagnosed glioblastoma, the integration of amino acid PET for surgical guidance remains supported by limited prospective data obtained from multiple centers. Beyond contrast-enhancing MRI, amino acid PET provides crucial biological insights into nonenhancing, invasive tumor margins, potentially optimizing the delineation of surgical boundaries.

On May 31, 2026, Cheng et al. published a study entitled “PCMDS: A New ^18^F-FET PET/MR-Based Tumor Resection Plan Improved the Prognosis of Glioblastoma Patients. A Multicenter Validated Study” (https://doi.org/10.34133/research.1335) in the journal *Research* (which is part of the *Science* Partner Journals program, published by the American Association for the Advancement of Science (AAAS), also the publisher of the journal *Science*).

This multicenter prospective trial involving 5 sites in China analyzed, in a matched-pair design, the added value of amino acid PET using the tracer *O*-(2-[^18^F]-fluoroethyl)-L-tyrosine (FET) versus conventional MRI alone for planning image-guided resection in 239 patients with newly diagnosed glioblastoma (according to the 2021 WHO classification) with regard to the extent of resection and overall survival.

Extent of resection was validated intraoperatively using ultrasound, intraoperative MRI, and ultramicroscopic cellular imaging. After surgery, all patients underwent radiotherapy with concurrent and maintenance temozolomide chemotherapy according to the EORTC 22981/26981 and NCIC CE.3 trial protocol.^1^

The results demonstrated that patients in the FET PET-guided resection group achieved a significantly greater extent of tumor resection and lower residual tumor volumes than the MRI-only group. According to the RANO resect classifier,^2^ which utilizes the postoperative tumor volume on MRI to quantify the extent of resection, the FET PET group achieved a significantly higher proportion of Class 1 (supramaximal resection) resections than the MRI-only group (50% vs 29%). This translated into significantly longer median progression-free survival (12.5 vs 9.3 months) and overall survival (19.7 vs 16.0 months) for patients in the FET PET group.

The present study bridges the gap between advanced imaging and definitive clinical survival endpoints using a rigorous, prospective multicenter design. Crucially, it establishes strong class 2 evidence—representing a major achievement in the field—which is largely lacking, as many earlier amino acid PET studies had a retrospective character and data were obtained predominantly from single-center studies^3^^,^^4^ with considerable heterogeneity.^5^

While amino acid PET imaging is traditionally viewed as a purely diagnostic tool (i.e. particularly for delineation of glioma extent, biopsy guidance, differentiation of treatment-related changes from tumor progression, and response assessment), incorporating amino acid PET into the surgical workflow may improve target delineation and reduction of postoperative tumor volume, and thus has the potential to extend overall survival. Remarkably, this image-guided surgical optimization achieves a survival benefit that surpasses many systemic anticancer agents evaluated in phase 3 glioblastoma trials.

Limitations of the study include the limited availability of intraoperative MRI and 5-aminolevulinic acid (5-ALA) was not utilized for resection guidance. On the other hand, intraoperative MRI as used here may specifically allow a more reliable assessment of the extent of resection, including residual tumor,^6^ also in the MRI-only group. Thus, any resulting improvements in residual tumor assessment and extent of resection would be expected to apply equally to the FET PET/MRI and MRI-only groups. Moreover, it has been shown to be non-inferior to 5-ALA fluorescence-guided surgery for achieving complete resections.^7^

In conclusion, the present study provides compelling evidence that FET PET-guided surgical planning could be considered an important, potentially practice-changing tool to improve the prognosis of patients with glioblastoma undergoing surgery. Hence, a prospective, randomized, multicenter study is needed to confirm these observations.

## Data Availability

Data sharing is not applicable to this article as no new datasets were generated or analyzed.
